# Bioactive Inorganic Materials for Dental Applications: A Narrative Review

**DOI:** 10.3390/ma15196864

**Published:** 2022-10-02

**Authors:** Khalid S. Almulhim, Mariam Raza Syed, Norah Alqahtani, Marwah Alamoudi, Maria Khan, Syed Zubairuddin Ahmed, Abdul Samad Khan

**Affiliations:** 1 Department of Restorative Dental Sciences, College of Dentistry, Imam Abdulrahman Bin Faisal University, Dammam 31441, Saudi Arabia; 2 UWA Dental School, The University of Western Australia, Crawley 6009, Australia; 3 College of Dentistry, Imam Abdulrahman Bin Faisal University, Dammam 31441, Saudi Arabia; 4 Department of Oral Biology, University of Health Sciences, Lahore 54600, Pakistan

**Keywords:** bioceramics, bioactive materials, hydroxyapatite, amorphous calcium phosphate, bioactive glass, calcium silicate, dental applications, dentistry

## Abstract

Over time, much attention has been given to the use of bioceramics for biomedical applications; however, the recent trend has been gaining traction to apply these materials for dental restorations. The bioceramics (mainly bioactive) are exceptionally biocompatible and possess excellent bioactive and biological properties due to their similar chemical composition to human hard tissues. However, concern has been noticed related to their mechanical properties. All dental materials based on bioactive materials must be biocompatible, long-lasting, mechanically strong enough to bear the masticatory and functional load, wear-resistant, easily manipulated, and implanted. This review article presents the basic structure, properties, and dental applications of different bioactive materials i.e., amorphous calcium phosphate, hydroxyapatite, tri-calcium phosphate, mono-calcium phosphate, calcium silicate, and bioactive glass. The advantageous properties and limitations of these materials are also discussed. In the end, future directions and proposals are given to improve the physical and mechanical properties of bioactive materials-based dental materials.

## 1. Introduction

The ultimate goal of restoring tooth structure and bone regeneration depends on using durable and biocompatible material. Bioceramics-based materials have been widely used in hard tissue repair and regeneration and recently gained interest in dental sciences [1]. Since the 1960s, bioceramic materials have been used in orthopedics in forms of oxides, such as aluminum oxide (A1_2_O_3_, alumina) and zirconium dioxide (ZrO_2_, zirconia) [2]. A screw-shaped dental implant made from alumina bioceramics, named Crystalline Bone Screw—CBS^®^, was first developed in 1962 and used in many cases [3,4]. A year after, another alumina bioceramic (Cerosium^TM^) was used as a bone substitute in case of large bone defects [5]. At that time, several orthopedic surgeons were also interested in using bioceramics for hip arthroplasty cases. The first total hip replacement with an alumina bioceramic material was performed in 1970 [6]. Soon after, another advancement in the field of alumina bioceramics was made by several German scientists, which resulted in the development of a number of ceramic orthopedic devices (BIOLOX^®^ alumina and BIOLOX^®^ delta alumina-zirconia ceramic composite) [7]. Oxide bioceramics are considered stable inorganic materials that are known for their chemical inertness and biocompatibility [8]. However, an inert ceramic does not form a bond with bone, and its artificial nature prevails [9]. Based on these, researchers focused on the usage of bioactive ceramics because these ceramics can react with physiological fluids forming biological-type apatite [10,11].

A report (Report ID: GVR-2-68038-840-4) (https://www.grandviewresearch.com/industry-analysis/bioactive-materials-industry) (accessed on 15 July 2022) was published related to market size, share, and trend analysis of the bioactive materials. It is reported that in 2020, the global market size of bioactive materials was approximated at USD 2.0 billion and was expected to increase at a compound annual growth rate of 14.0% from 2021 to 2028. Among bioactive materials, glass-based materials led the market with a 30% global revenue share. It was interesting to find in the report that the dentistry applications led the market with a 45% share of the global revenue. The commonly used bioactive ceramics or bioactive inorganic materials were amorphous calcium phosphate (ACP), hydroxyapatite (HA), tricalcium phosphate (TCP), tetra-calcium phosphate (TTCP), monocalcium phosphate (MCP), dicalcium phosphate (DCP), calcium silicate (CS), and bioactive glass (BAG) [12,13]. Among the calcium-phosphate family, there was variation in the calcium phosphate (Ca/P) ratio, whereby the resorption of these materials depended on the Ca/P ratio [14]. If Ca/P was less than 1, the ability to reabsorb increased. The resorption of the calcium phosphate family depended on the crystallographic structure, size of the particles, and density [15]. The apatite structure could be preserved with Ca/P ratios as low as 1.5; therefore, HA with a lower than normal ratio (1.67) was characterized as calcium-deficient or non-stoichiometric [16].

**Figure 1:**
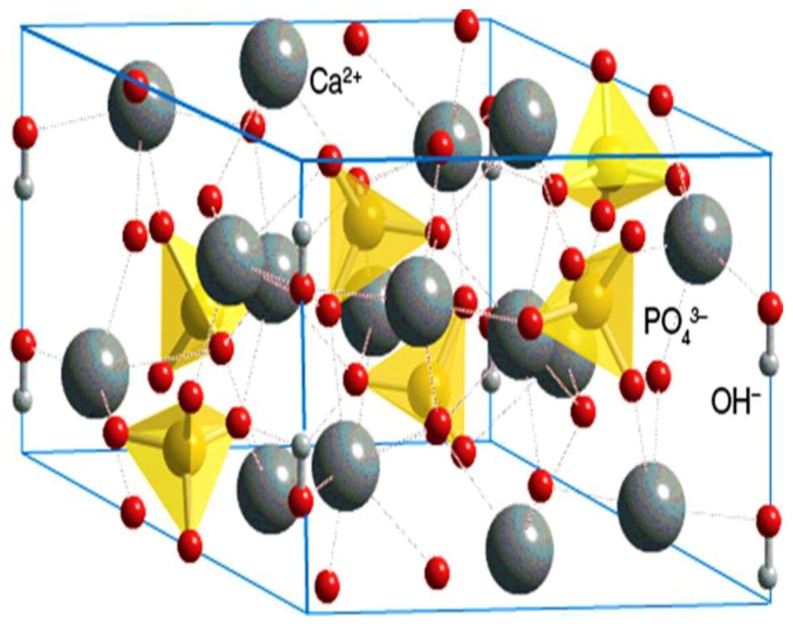
Schematic hexagonal structure of the hydroxyapatite, Ca = calcium; PO_4_^3−^ = phosphate; OH = hydroxyl [19].

These bioactive materials are biocompatible, non-inflammatory, non-irritant, and non-toxic, with the benefit of showing bioactivity at an interface with natural tissue [17]. The natural hard tissues (bone and teeth) consisted of apatite or apatite calcium phosphate. The chemical analysis of enamel, dentin, and bone exhibits calcium and phosphate as principal components, whereas the inorganic phase of bone and teeth was calcium-deficient HA [12]. Biological apatite has certain differences from pure HA and is a poorly crystallized structure. It is important to know the structure of biological apatite and how closely these synthetic materials resemble natural materials. The schematic structure of the HA is presented in Figure 1. The composition, crystal size, morphology, and stoichiometry of biological apatite differ from pure HA [18]. The calcium/phosphorous (Ca/P) molar ratio was 1.67 for pure HA, while for enamel and dentin it was 1.62 and 1.64, respectively.

**Figure 2:**
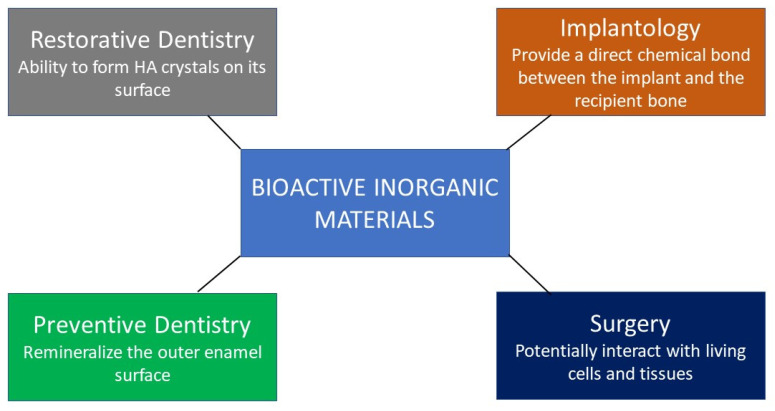
Perception of bioactive materials and their functions in different clinical applications.

The dental biomaterial sciences have been evolving, and there is a paradigm shift in the development of dental materials, whereby there is shifting balance towards regenerative materials (resorbable) or bioactive materials with specific requirements. Such materials could be tailored for enhanced adhesion, quicker healing, and fast tissue regeneration. A recently published bibliometric study [20] showed the trend of dental biomaterials research. It was mentioned that there was a significant increase (55.52%) in research related to bioceramics from 2012 to 2019, compared to 2007 to 2011. The availability of these bioactive dental materials influenced dental practices and treatment planning [21]. The bioactive materials have gained significant importance in the last two decades for the management of tissue loss due to dental caries, periodontal infection, and bone defects [22,23,24]. The release of ions from the bioactive materials elicited an effect and increased the chances of regeneration and remineralization. Various studies have been conducted to assess the bioactive potential of biomaterials in the oral environment and evaluated their performances in multiple clinical applications [25,26,27,28]. The outcomes of these studies showed that the bioactive materials’ applications to enhance oral health were developing at a rapid pace. Much research in relation to dentistry has been conducted based on HA, TCP, calcium phosphate, bioactive glasses, and calcium silicate [29,30,31]. However, it is important to note that the perception of “bioactive” differs with the clinical application [32], as shown in Figure 2.

In the last decade, many review articles focused on bioactive materials and their applications in dentistry. However, the focus was more on specific applications, such as endodontics [33,34], regeneration [35,36], restorative dentistry [37,38], and implantology [39,40]. Therefore, to fill this missing gap, this present narrative review aims at providing a comprehensive overview of dental applications of bioactive inorganic materials. For a better understanding, special focus is given on the structure of bioactive inorganic materials and their clinical applications, such as restorative dentistry, endodontics, periododontology, surgery, and implantology. For this review, the articles were searched through Google Scholar, PubMed, Scopus, and Web of Science, whereby the focus was given on the laboratory and clinical trial studies. The inclusion criteria for the narrative review article were:

(i) Use of bioactive inorganic materials i.e., amorphous calcium phosphate, hydroxyapatite, tri-calcium phosphate, bioactive glass, and calcium silicate for dental applications, including restorative dentistry (adhesives and fillings), endodontics, periodontology, implantology, and surgery.
(ii) Bioactive inorganic material’s studies included lab-based analysis, in vitro, in vivo, and clinical trials.

The exclusion criteria were:

(i) Studies not published in indexed journals.
(ii) Grey literature and articles published in non-English language.

## 2. Bioactive Inorganic Materials

### 2.1. Amorphous Calcium Phosphates (ACP)

**Figure 3:**
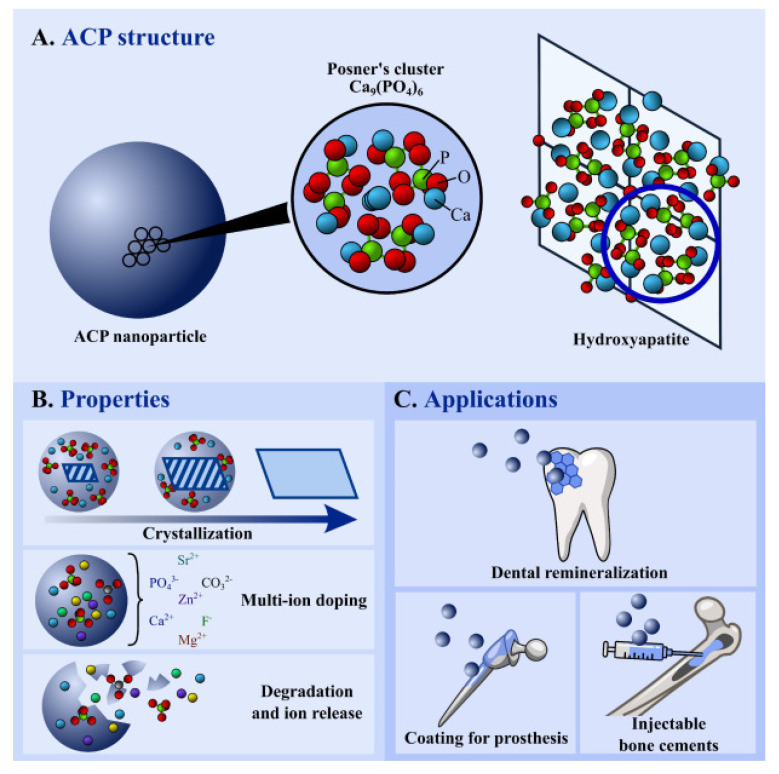
(**A**) Structure of ACP, and the relationship with HA unit cell, (**B**) properties, and (**C**) biomedical applications [44].

In 1870, Dr. Junius E. Cravens (USA) used calcium orthophosphate powder and lactic acid liquid on the pulp tissue to maintain the pulp vitality [41]. In the 1960s, ACP [Ca_9_(PO_4_)_6_] with a molecular weight of 310.174 g.mol^−1^ was first reported by Aaron S. Posner. Dr. Posner mixed high concentrations of sodium acid phosphate and calcium chloride, which led to the amorphous precipitation of calcium phosphate (Figure 3A) by accident [42]. After mixing calcium and phosphate, spontaneous amorphous precipitation (Ca/P molar ratio~1.50) occurred, which after some hours, led to the appearance of crystalline apatite (Ca/P molar ratio~1.67) [43]. This unique property, with respect to crystalline apatite, was due to its metastable nature (Figure 3B), and it had a much higher degradability than any other calcium phosphates [44]. Based on these properties, it was one of the most widely reported materials in biomedical applications (Figure 3C).

The ACP was readily converted into crystalline HA, which was desirable during hard tissue maturation. The crystalline HA was more acid-resistant and had better mechanical properties. ACP had shown better cell adhesion with hard and soft tissues, biocompatibility, bioactivity, and osteoconduction properties [45]. It increased cell proliferation activity by increasing alkaline phosphatase activity. ACP was one of the bioactive materials applied to repair and regenerate bone tissues in dental surgery. There was evidence that the precursor phase of ACP could be deposited through intracellular vesicles that contained minerals at the gaps that may have been apparent in the collagen matrix of dental bones [46]. Based on its bioactive properties, the ACP was applied as a transient phase that could be readily utilized as a precursor in apatite growth.

The biomineralization of ACP was affected by many proteins, enzymes, and ions, such as dentin matrix protein [47]. It was reported that the biomineralization of calcium phosphate crystals was controlled by alkaline phosphatase (ALP), osteocalcin (OCN), and osteopontin (OPN), whereby ALP supported mineral formation. However, OCN and OPN (most abundant non-collagenous proteins) were both inhibitors of mineralization [48]. It was also reported that ACP had superior biodegradation and osteoconduction than other members of the calcium phosphate family, such as TCP and HA [42]. ACP had the potential for continuous delivery of constituent ions over an extended time span, leading to the emergence of stable product bioapatite. Therefore, it was an intermediary product during the synthesis of HA [49]. The ability of metastable and relatively soluble synthetic ACPs to release calcium and orthophosphate ions in the acidic oral environment allowed ACP to potentially participate in the remineralization of dental enamel [50,51,52,53,54]. ACPs were discovered to exhibit less hardness and elastic modulus than HA [55,56], whereas it showed comparable fracture toughness to single crystals HA, but nano-grained HA had a higher fracture toughness [57]. The mechanical properties of the crystals were directly related to the amount of calcium ions within the crystal. The limitation associated with ACP was due to existence of several distinct amorphous phases with different chemical composition, local order, and physiochemical behavior [58].

### 2.2. Hydroxyapatite (HA)

**Figure 4:**
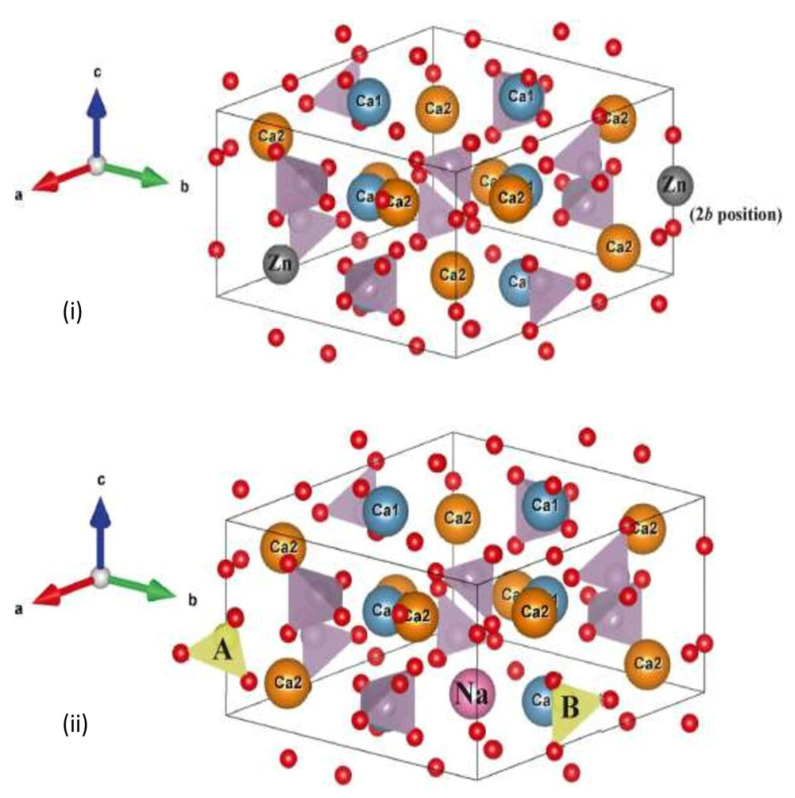
The crystal structure of (**i**) zinc-substituted hydroxyapatite, where the substitution of Zn on Ca2 site and at *c*-axis in the 2*b* position between two oxygen ions; (**ii**) A- and B-type carbonate ion substitution in hydroxyapatite structure, Ca = calcium, Na = sodium; Zn = zinc [62].

Despite a wide range of compositions, the ‘apatite—the most stable form’ belongs to a family of compounds sharing similar structure in vivo [59]. HA has a hexagonal structure with a Ca/P molar ratio of 1.67 and a molecular weight of 1004.6 g/mol. HA depicts the accurate crystallographic orientation and location of atoms in the unit cell. Further, it is a compound with a definite crystallographic structure and composition [60]. This crystallographic form allows the substitutions of many other ions to gain optimum dual benefits without changing its symmetry [61]. Various anionic, as well as cationic substitutions, can be made within the crystal lattice of HA (Figure 4), producing no or minimal adverse effects on the biological system when implanted [62].

The carbonate group, which substitutes phosphate (PO_4_)^3−^ or hydroxyl (OH)^−^, is called type A or type B substitution, respectively [63,64], and with this substitution, the crystal lattice structure alters [65]. The synthetic carbonate substituted HA is expected to have enhanced biocompatibility [66]. The enhanced cellular activity and enzyme production after implantation dropped the biological pH at the implant site, which initiated the HA’s resorption and subsequently released Ca^2+^, HPO_4_^2−^, and PO_4_^3−^ ions. This ionic release thus supersaturated the local environment with respect to calcium and phosphate, as well as changed the surface chemistry of HA particles. Under these conditions, a layer of carbonated HA tended to form over the HA crystals through seeded growth [67]. The substitution of chloride (Cl) changed the hexagonal system into a monoclinic one to accommodate alternating large Cl ions. Substituting fluoride (F) for OH usually increased crystallinity, which was often associated with contraction, increased size, and stability [68]. The surrounding biological tissue i.e., extracellular matrix (ECM), of the biological system could tolerate only optimized and appropriate amounts of these substituted species within ceramic apatite [69]. Ionic substitutions brought about changes on the surface of apatite, modifying its surface structure and surface charge. The surface of apatites was known to have a very influential role in the response toward biological environment upon implantation [70].

**Figure 5:**
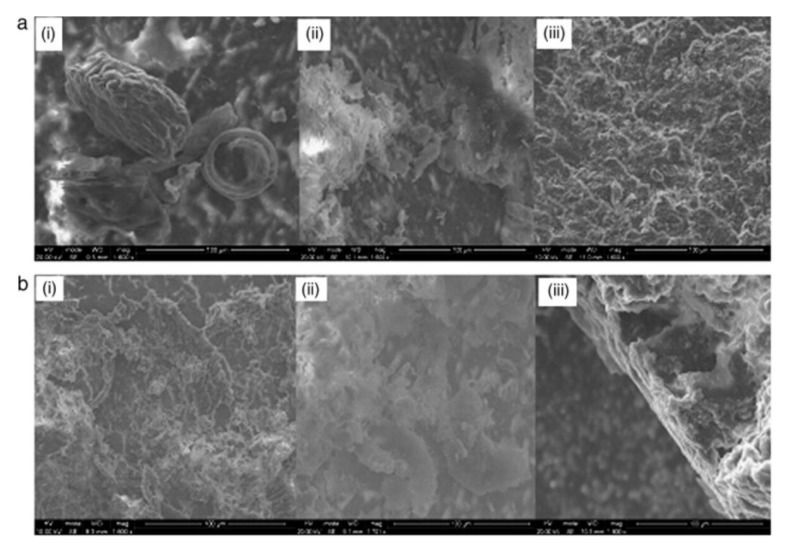
SEM images of polymer/fluoride-substituted HA composites-tooth interface after immersion for 90 days in (**a**) deionized water (**b**) artificial saliva. The images were taken after the push-out test. The concentration of fluoride-substituted HA varied i.e., ((i) 10%, (ii) 15%, and (iii) 20%) [77].

HA was classified as bioactive, referring to its support towards the formation of hard tissue and osseoconduction when used in orthopaedics, maxillofacial applications, infra-bony defects, ridge augmentation, hip and joint prosthesis, and a well-known antidote to a tooth’s demineralized enamel or dentin, and lastly in dental implant coatings [71,72,73]. There are many contributing factors, including composition, structure, size, shape, surface area, and density, which can affect the kinetic process of new apatite formation on the surface of HA. It was anticipated that the presence of negative charges (hydroxyl and phosphate) on the surface of HA could interact with positive charges (calcium), subsequently forming a calcium-enriched amorphous calcium phosphate (ACP). The increase of positive charges on the HA surface interacted with the negative phosphate ions to form a calcium-deficient ACP. This was considered as brushite, which eventually crystallized into bone-like apatite [74,75]. Khan et al. incorporated fluoride substituted (nano-fluoroapatite) in the polymeric network, synthesized dental composite, and restored prepared tooth cavity to determine the bonding behavior after immersion in deionized water and artificial saliva. It was found that after placement for 90 days in media, new apatite formation occurred on the tooth surface, as shown in Figure 5. The formation of apatite was expected, due to the release of calcium and phosphate from the material. The development of the apatite layer may have a complex influence due to biomaterial’s chemical structure and composition, pore structure, size, and volume [76]. It was revealed that fluoroapatite had a minimal tendency to release fluoride, even after six months [77].

The anisotropic behaviors of HA are mainly limited to protein dissolution, absorption, crystal growth, and interfacial bonding mechanism. It can be the epitaxial/non-epitaxial bonding of bone apatite to HA [78]. A study reported the synthesis of nano-apatite using a microwave irradiation technique and heat treated at different temperatures. Later, they investigated the cytotoxicity and interaction of prepared nano-apatites with HeLa and SF767 cell lines periodically for up to 7 days. It was found that nano-apatite particles tended to show a proliferation/attachment of cells [79], as pure HA had a low dissolution rate and pure TCP had a very high dissolution rate. Therefore, biphasic apatites (HA/TCP) could affect the dissolution tendency and bone-bonding [80]. The reported dissolution order of calcium phosphate members was: TTCP > α-TCP > DCPD > DCPA > OCP > β-TCP > HA [45]. The dissolution process of the calcium phosphate family also depended on the crystal structure, crystal dimensions, surrounding pH, and ionic strength [81]. Although HA had many advantages, however, limitations such as poor mechanical properties were associated with it. It could not withstand tensile forces in bulk, due to its low impact strength and fracture toughness [82]. The poor mechanical properties were due to the porous structure, which could be enhanced by including whiskers, fibers, and nanoparticles [83,84,85].

HA nanoparticles promoted enamel surface remineralization, reduced dentinal hypersensitivity, and had been studied in preventive dentistry [86,87]. When compared to ACP-based materials, HA was more effective in increasing the calcium and phosphate content of enamel, and this effect was more pronounced over a longer treatment period [88].

### 2.3. Tricalcium Phosphates (TCP)

**Figure 6:**
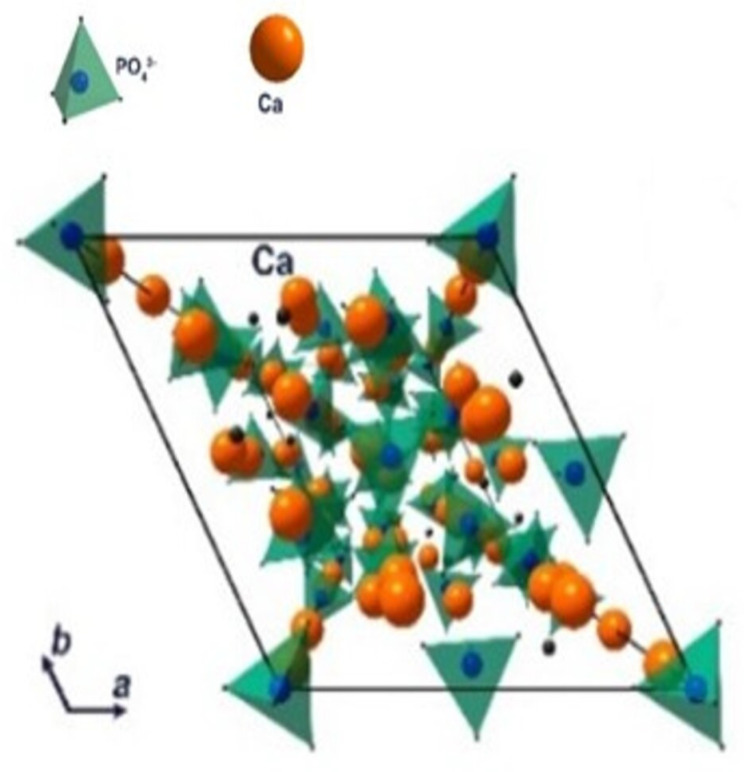
Schematic structure of β-TCP Ca = calcium; PO_4_^3^^−^ [94].

In 1920, tricalcium phosphate (TCP) was first described by Albee when he successfully repaired a human bone defect with TCP [89]. TCP exists in three polymorphic forms, the ultra-high temperature α’-TCP, high-temperature α-TCP, and the low-temperature β-TCP [90]. The α-TCP only exists at very high temperatures (around 1500 °C) and is not generally used. β-TCP is unstable at high temperatures and changes into α-TCP (1125–1200 °C) and reverts to the original single β phase if it cools properly. α-TCP is safe at high temperatures and single-phase powder can be achieved successfully [91]. Both β-TCP and α-TCP have the same constituent elements, Ca_3_(PO_4_)_2,_ with a Ca/P ratio of 1.50 [92]. The crystalline unit cell of both α- and β- TCP comprises Ca^+2^ and PO_4_^3−^ ions. The different crystallographic orientation of β-TCP (hexagonal) and α-TCP (monoclinic) leads to different characteristics [93]. The schematic structure of β -TCP is given in Figure 6.

**Figure 7:**
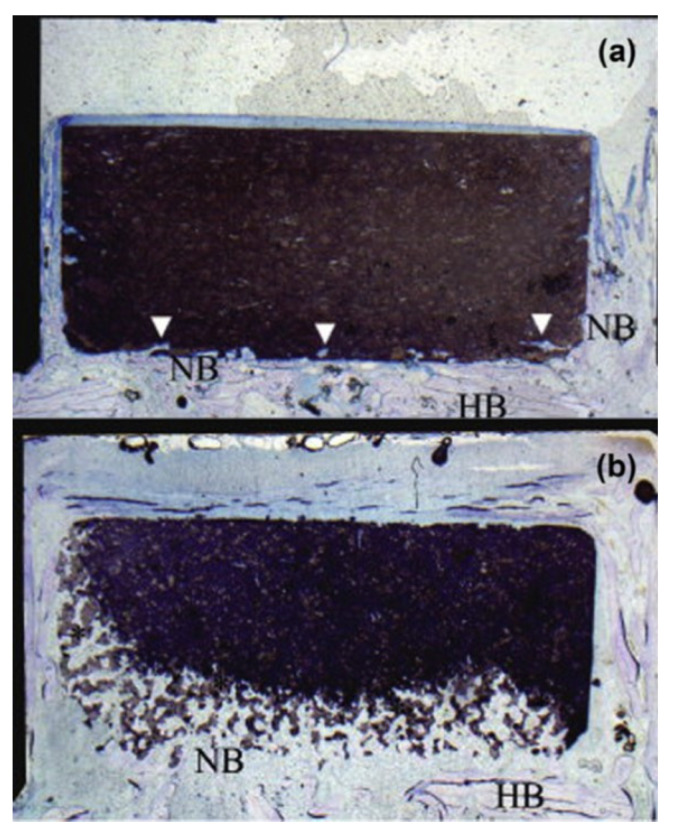
The image showing degradation behavior of (**a**) β-TCP and (**b**) α-TCP block in the calvaria region of rabbits after 8 weeks implantation. It was found that the α-TCP block showed considerably more degradation than the β-TCP [105]. NB = new bone; HB = host bone.

The β-phase stabilizers are zinc, strontium, and magnesium, while the α-phase stabilizer is silicon (Si), which replaces PO_4_ [95]. TCPs are bioactive, biocompatible, osteoinductive, and bioresorbable materials that have the ability to allow and promote bone tissue regeneration. It was being used as an absorbable material in the development of tissue engineering scaffolds [96]. β-TCP had found more uses in clinical applications comparatively, and was also used in developing monophasic/biphasic bioceramics [97,98,99,100,101]. However, composites containing α-TCP were common in repairing bone defects [60,102]. A study compared the α-TCP and β-TCP as bone graft material in the calvaria region of Japanese white rabbits for 2, 4, and 8 weeks. No significant difference was found after two weeks; however, a significant difference was observed after 4 and 8 weeks. α-TCP showed more degradation at 4 weeks compared to β-TCP, and severely degraded after 8 weeks as shown in Figure 7. For bone void filling and bone regeneration, TCPs had been used in dental surgery [103]. However, due to their poor mechanical properties, low cohesion, brittleness, and no macroporosity, their clinical applications were limited to non-loadbearing applications [96,104].

### 2.4. Bioactive Glass

**Figure 8:**
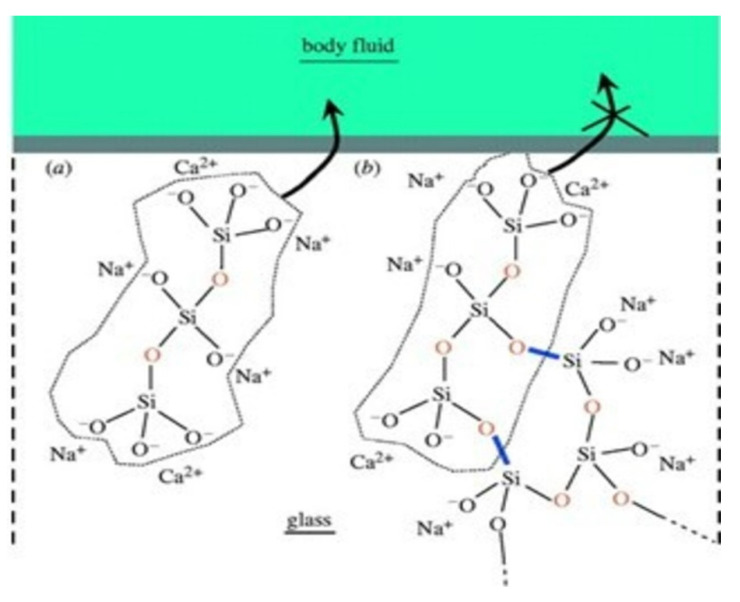
The chemical structure of bioactive glasses; (**a**) showing no linkage with the adjacent structure, where (**b**) showing cross-linked structure, Ca = calcium; Na = sodium; O = oxygen; Si = silicon [110].

In 1969, Larry Hench and co-workers laid the first stone of bioactive ceramics by bringing chemically bone-alike Hench’s 45S5 Bioglass^®^ into the market [106]. This first bioglass was based on 45% SiO_2_, 24.5% Na_2_O, 24.5% CaO, and 6% P_2_O_5_, tested in a rat femoral implant. After a successful six weeks of implantation, Larry Hench published his results in 1971 and opened a new horizon of bioceramics [107]. The schematic structure of bioactive glass is given in Figure 8. The first generation of bioglass was biologically inert, while the second generation of bioglass concept changed from bioinert to bioactivity. The third generation led to the development of resorbable bioactive bioglass [108]. The mechanism of bone formation by bioactive glass was comprehensive yet simple. In the first hour after implantation, Si-OH bonds developed, leading to the release of Si(OH)_4_ ions in the surrounding area. In the next hour, Si(OH)_4_ reacted to form a hydrated mesh of silica gel, and after 24 h, Ca, PO_4_, and CO_3_ precipitated on the top layer of silica gel leading to the foundation of carbonate apatite. Later, macrophages and differentiated stem cells accumulated in the carbonated apatite leading to the formation of a bony matrix. Finally, the matrix crystallized, and bone growth was enhanced [109].

There are different types of bioactive glass, such as the traditional Hench’s Bioglass^®^ and it was used as a bone substitute material [111]. Later, phosphate-based bioglass and borate-based bioglass were introduced [112,113,114]. Recently, bioactive glass particles, micro-, and nano-sizes had been introduced, combined with polymers, to form a composite for diverse applications [115,116,117,118,119]. 45S5 was the traditional bioglass, while later other types such as BG605 and S53P4 were introduced; these bioactive glasses showed a deposition of collagen, bone deposition, and regeneration. Moreover, they showed bacteriostatic properties [120,121,122,123]. Many in vitro and in vivo studies had reported better bonding of bioactive glass with the bone than all other bioceramics, whereas in clinical practice calcium phosphates were more commonly used [82,124,125,126,127,128,129]. Nonetheless, many obstacles, shortcomings, and frontiers must be surpassed to make these biomaterials beneficial to patients.

### 2.5. Calcium Silicate

Calcium-silicate-based bioceramics are commonly used in various load-bearing biomedical applications due to their innate biocompatible nature. They also possess chemical similarities with bones and osteoconduction properties [130]. It was introduced in 1970 as hard tissue replacement [131]. There were two crystalline modifications of stoichiometric calcium silicate (CaSiO_3_), i.e., low-temperature modified triclinic wollastonite (β-CaSiO_3_) with a space group of α*P*30, and monoclinic pseudo-wollastonite (α-CaSiO_3_) with a space group of *C2*/*c*. Dicalcium silicate (Ca_2_SiO_4_) had four different modifications: γ-phase, which was stable at room temperature; α′- and α-phases, which were stable at high temperatures; and β-phase, which was not thermodynamically stable. Tricalcium silicate (Ca_3_SiO_5_) was a phase-stable between 1250 °C and 1900 °C [132].

**Figure 9:**
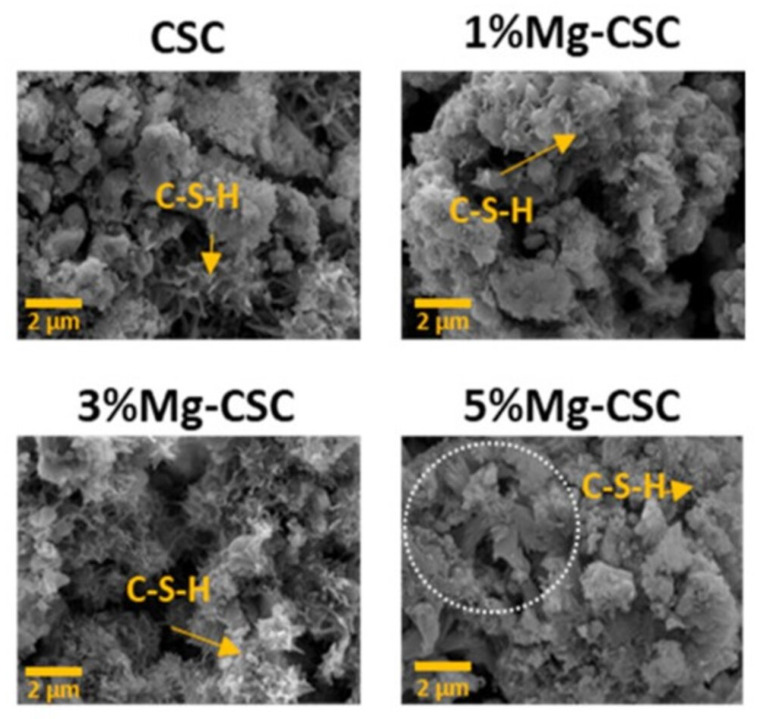
SEM images of calcium silicate and co-synthesized 1%, 3%, and 5% Mg-calcium silicate (Mg-CSC) cements after immersion in simulated body fluid (SBF) for 21 days; all samples presented the foil-like C-S-H aggregates (yellow arrows); 5% Mg-CSC showed hydroxyl-magnesium-silicate minerals [139].

Previous data revealed calcium silicate bonded with living bone and soft tissue by forming an apatite layer on its surface [133]. Its main drawback was low fracture toughness and hardness, which limited its use in implant application, however, it could be improved by the addition of alumina [134]. Aluminum-free calcium silicate had been proven useful in endodontic treatment due to less setting time. It had been used in pulp capping, cavity lining, root canal obturation, etc. [135]. It had the ability to induce differentiation of cells into osteogenic cell lineages due to a property known as osteoinductiveness [136], which could be due to the release of calcium ions. It was reported that the release of calcium supported the differentiation of dental stem cells and enhanced the biomineralization process [137]. With all the advantages of biocompatibility, limitations were associated with calcium silicates, such as high brittleness and insufficient bioactivity, compared to calcium-phosphate based materials. To overcome these limitations, other components such as magnesium (Mg), zinc (Zn), and zirconium (Zr) could be added to form ternary compounds. The addition of these components could not only improve the mechanical properties of the calcium-silicate materials, but also maintain the bioactive properties, as shown in Figure 9 [138,139].

Calcium silicate-based dental biomaterials have been commercialized and are composed of di/tri-calcium silicate phases such as ProRoot MTA (Dentssply Sirona, Bensheim, Germany), Biodentine (Septodont Inc., Saint-Maur-des-Fossés, France), MTA Flow (Ultradent, South Jordan, UT, USA), Ortho MTA (DO Co. Ltd., Seoul, Korea), BioRoot RCS (Septodont Inc., Saint-Maur-des-Fossés, France), iRoot SP (Innovative BioCeramix Inc., Vancouver, BC, Canada), TotalFill (Innovative Bioceramix Inc., Vancouver, BC, Canada), EndoSeal (Maruchi Co. Ltd., Daegu, Korea), etc. [140,141].

## 3. Clinical Applications of Bioactive Inorganic Materials in Dentistry

All bioceramics have diverse dental applications in restorative, endodontics, implantology, and periodontal fields of dentistry [105,142,143]. The following sections discuss the clinical applications of various inorganic bioactive materials.

### 3.1. Restorative Dentistry Application

**Figure 10:**
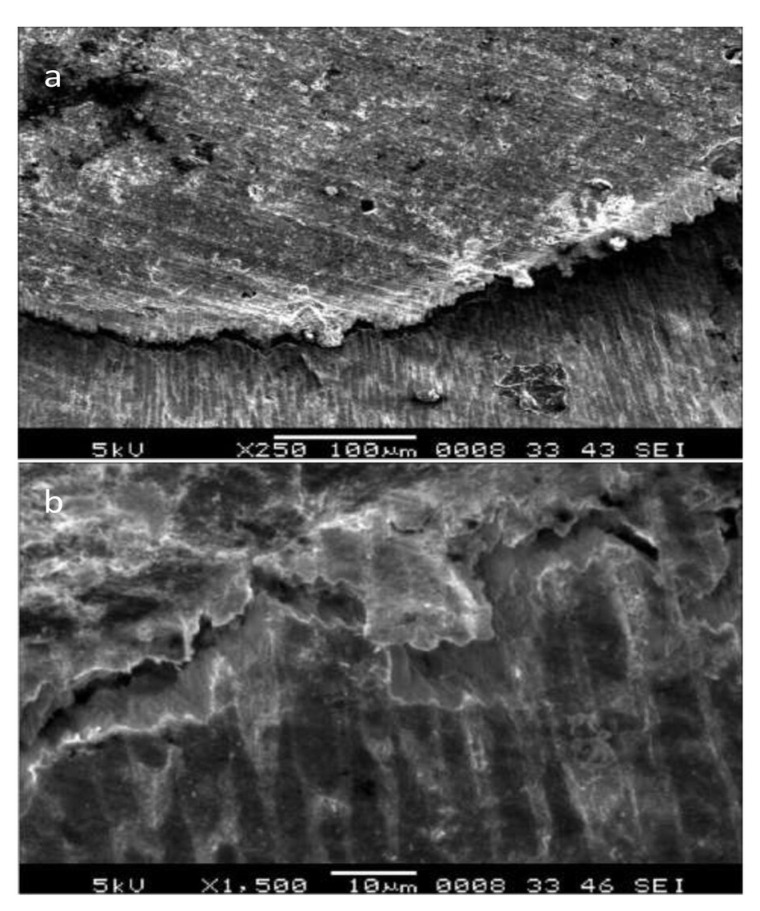
SEM images of a buccolingual section showing (**a**) a white zone at the tooth surface-sealant interface and (**b**) a white irregular granular zone at the tooth surface-sealant interface [151].

The ACP-based composites with remineralization potential have been employed in minor dental caries defects to seal pits and fissures, and in teeth where plaque accumulation led to bacterial infection [144,145]. These ACP powder or particle-based materials could aid in preventing the demineralization of tooth enamel when added to orthodontic adhesives [146,147] to lower the risk of formation of white spot lesions [148]. Desirable properties could be achieved by combining these with adhesives; however, the Food and Drug Administration (FDA) approval demanded these agents be categorized as drugs, leading to hampered marketing. A product such as Recaldent (Recaldent Pty. Ltd., Melbourne, Australia) was approved by the FDA, in which ACP was one of the ingredients [149]. A study proved the efficacy of photo polymerized ACP in Aegis-Ortho (The Bosworth Co., Midland, TX, USA) and found its use as a potential bonding agent [150]. A study [151] showed the remineralizing potential of ACP containing (Aegis—Opaque White, Bosworth Co. Ltd., Skokie, IL, USA) on the tooth enamel surface. The teeth were challenged under the pH cycle for two weeks. The results showed the appearance of a white zone at the tooth surface-sealant interface and a more-zone was observed in the region of fissures with increased irregularities. The white irregular granular was observed and considered as new apatite crystals, as shown in Figure 10.

The anti-demineralizing/remineralizing ACP composites could be used as base materials under permanent dental restorative materials in patients who were more vulnerable to dental caries due to multifactorial reasons [152]. However, the main drawback of ACP is its incapability to enhance the value of resin-based dental composites mechanically, as compared to silanized glass fillers [153]. Due to its unacceptable low flexural strength, it could not be used as a permanent posterior tooth restorative material where masticatory force is maximum. Therefore, there is a need to develop bioactive fillers that are mechanically strong enough to bear the load during mastication for a wide range of tooth restorations without failure [154]. Furthermore, the main problem is the cluster formation of ACP particles, leading to the low potential to fight crack initiation and propagation and ultimately low interfacial strength comparatively [155]. Hence, more focus should be paid to the homogenous particle distribution by controlling the parameters governing the size and surface properties of ACP [156].

The mechanical properties of ACP-based dental materials were improved when combining ACP with glass particles, whiskers, and calcium fluoride (CaF_2_) nanoparticles [157]. Xu et al. patented and used nano-amorphous calcium phosphate (NACP; 5–90 wt.%) particles (10–500 nm) in resin-based composite, and they found similar antibacterial and mechanical properties to commercial composites [158]. Zhang et al. [159] brought rechargeable calcium phosphates composites into the market for the first time using NACP, which proved its potential by inhibiting carious lesions through Ca and P ions’ continuous delivery and recharging capabilities over a long period. It was also reported that ACP in the lining and luting agents had a detrimental effect on the compressive strength, while showing increased diametral tensile strength. When it came in contact with the oral aqueous environment, it caused a loss of calcium and phosphate minerals, causing weakening of the dental composite; however, dental composites based on HA did not show this behavior [157,160].

Crystallized HA in dental composites was first used by Okazaki and Ohmae [161]. They mixed these fillers with 2.2′(4-methacryloxydiethoxyphenyl) propane and found improvement in the mechanical properties, higher apatite concentration in resin matrix with a plateau above an apatite-resin ratio (Ap/R) of 1. Santos et al. [162] incorporated silanized and non-silanized HA in the resin matrix and reported increased mechanical properties (elastic modulus and flexural strength). Arcis et al. [163] investigated microscopic and nanoscopic HA fillers in dental resin matrices. Interestingly, they surface-treated these filler particles with different coupling agents. Nanoscopic HA was surface treated with citrates, whereas microscopic HA was treated with non-concentrated malic or citric acid solutions and concentrated methacrylic or acrylic acid solutions. The concentration (wt.%) of microscopic and nanoscopic HA in resin matrices was 60% (35 *v*/*v*%) and 50% (26 *v*/*v*%), respectively. It was revealed that the surface hardness and elasticity modulus were increased in the experimental composites with the incorporation of both surfaces treated and untreated with HA, excluding malic acid-based materials. Domingo et al. [164,165] claimed that nano-sized HA filler-based composites were unsuitable for clinical performance and were hydrolytically not stable compared to composites with microscopic particles.

**Figure 11:**
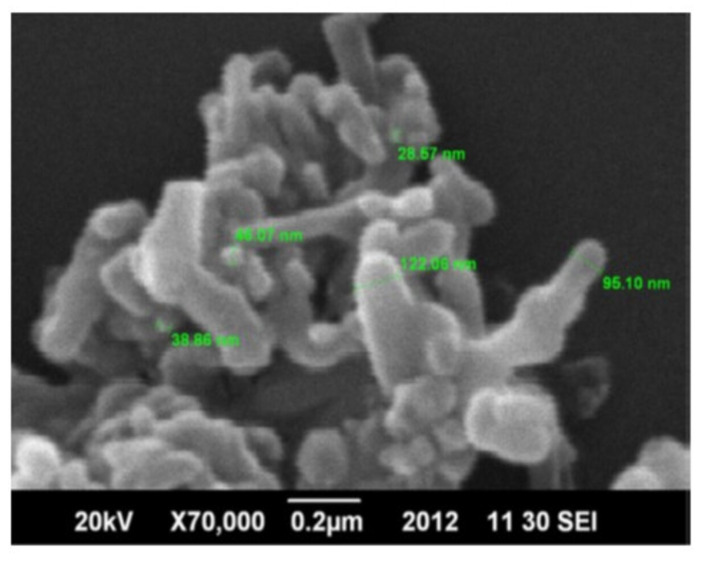
SEM image showing the nanostructure of hydroxyapatite prepared by the microwave irradiation technique [166].

In contrast, Lung et al. [166] synthesized nHA (Figure 11) and compared nHA silanized particles with non-silanized nHA-based experimental dental composites with commercial composites. A fair reduction in the water-sorption and the surface micro-hardness values were increased. The experimental nHA-based dental composites revealed that the surface micro-hardness was directly proportional to the filler concentration. Although the result was not significantly different; silanized nano-HA filler particles-based composites showed a higher surface hardness than non-silanized and commercial composites. The high surface hardness values of silanized-HA based composite could have been due to the better interaction of silanized particles with the polymeric network and it also showed less water sorption.

The filler particle size, type, and concentration affected the mechanical and physical properties, along with the resin matrix type (hydrophilic or hydrophobic) [167,168]. In another study [169], nano-HA rods were synthesized by using a hydrothermal technique and incorporated these as-synthesized nano-rods in an experimental adhesive containing ethanol, bisphenol glycol methacrylate (bis-GMA), urethane dimethacrylate (UDMA), hydroxyethyl methacrylate (HEMA), and 2-Ethyl-2-(hydroxymethyl)-1,3-propandiol trimethacrylate (TMPTMA). The concentration of nano-rods was 0.2%, 0.5%, 1%, 2%, and 5% by weight, in which they investigated the mechanical performance of these experimental adhesives. Their results revealed that the addition of fillers only up to 0.2–0.5 wt.% enhanced the strength and further increased in the filler concentration, which reported a steep decrease in mechanical behavior. The reduction in strength could be attributed to the cluster formation of filler particles after a certain percentage of filler content and/or due to incomplete polymerization of the bonding agent. Other factors also played a role, including the different refractive indexes of the organic and inorganic parts of dental composite and the irradiation wavelength [170]. HA addition in higher concentrations to the adhesives and bonding agents/restorations adversely opposed the optimum polymerization rates, due to the opacity of HA against visible light.

Chen et al. [171] synthesized nano-HA fibers by wet chemical precipitation and added these to dental resins (bis-GMA and TEGDMA) with variations in concentrations. The mechanical testing (biaxial flexural strength) was investigated, and it was revealed that the addition of 10 wt.% HA improved the flexural strength by 22.2%. However, the additional increase in the nano-HA fibers’ mass fractions (20% and 40%) did not enhance, but rather decreased the mechanical behavior. A similar pattern was observed when nano-HA and micro-silica particles were mixed together. The biaxial flexural strength of the experimental dental composite (3 wt.% nHA + 57 wt.% silica) was significantly higher (29.2%) when compared to the control group. The dual response of reinforcement and weakness could be attributed to the homogenous distribution or cluster formation behavior of nHA filler particles. However, a higher mass fraction of nano-HA fibers tended to form bundles that could not effectually strengthen the composites and subsequently deteriorate the mechanical properties of composites. In a comparative study, silanized HA whiskers and nano-powders were added to resin monomers separately, and the results favored whiskers over nano-powders in terms of the improvement in mechanical behavior [172].

In another comparative research conducted on nano-sized HA whiskers based dental composite and commercially available composite, the results revealed that HA whiskers with 20 vol.% had comparable properties. Until this range, better interaction was observed at the matrix/filler interface [173]. Zhang et al. [174,175] reported that a small fraction (up to 7 wt.%) of commercial HA could improve the curing capabilities and rate. Khalid et al. [176] compared experimental synthesized silanized nano-HA-based composites with flowable/micro hybrid dental composites. They disclosed the non-toxic behavior and least monomer elution with better polymerization value in the experimental composite. Khan et al. [177,178] synthesized nano-HA and covalently linked it with the backbone of polymers and proposed its potential application for obturating material. The mechanical and biological properties showed satisfactory results, and the bioactive composite showed the appearance of a new apatite layer on the tooth surface after immersion in artificial saliva. In a study, the mechanical properties of nHA were improved by using an in situ synthesis method to incorporate carbon nanotubes (CNT) with nHA. After silanization, the nHA/CNT was incorporated into dental resins, and the results showed improved mechanical properties and excellent biocompatibility in the CAM assay study [179]. The biocompatibility or cytotoxicity of the resin-based dental restorations mainly depended on the release of reactant monomers. Most of the monomers (TEGDMA, HEMA) released in first 24 h and could cause toxicity. Other components, such as photoinitiator (camphorquinone) had the tendency to show cytotoxicity. However, factors other than cytotoxicity, including immune, gene, or tissue reactions, could also play a role in determining the biocompatibility of the material [180]. The International Organization for Standardizations (ISO) specified the testing standards for the biocompatibility of the dental materials. ISO 7405 was specifically for dental materials, whereas ISO 10993 was for medical devices including dental materials. The biological testing methods for dental materials had been reported in detail [181]. The recommended tests for in vitro cytotoxicity analysis [182] were; (i) Direct cell culture and culture extract testing, or barrier screening assays; (ii) Agar diffusion testing; (iii) Filter diffusion testing; (iv) Dentin barrier testing.

In a study [68], basic mono- and reactive acidic and TCP fillers (MCPM/β-TCP) had been incorporated collectively in resin monomers. Due to the presence of MCPM, a higher water-sorption occurred; however, with the addition of β-TCP, the control of water-sorption and ion release was observed. The immersion in simulated body fluids resulted in reprecipitation of a mixture of brushite and HA on the surface of the sample, and it was suggested that increasing the concentration of MCPM, i.e., higher powder/liquid ratio could enhance the remineralization potential [183]. In a similar study [76], both MCPM/β-TCP were incorporated (0, 50, 60, and 70 wt.%) in dental resins along with antibacterial agents (chlorhexidine). The experimental composites were immersed in simulated body fluid, and it was reported that incorporating the reactive fillers increased apatite formation in proportion to CaP content. The mechanical properties (flexural strength) were decreased with the increased concentration of CaP contents, in which the lowest value recorded was around 100 MPa. In another study, the same group proposed [184] that water-sorption capacity was directly proportional to the filler content due to the hydrophilic nature of MCPM. However, the degree of conversion of these experimental composites (78%) was improved compared to the commercial composite (55%). In another study, tri-strontium phosphate was added with MCPM and polylysine, and these components were incorporated in resin monomers. A new apatite layer was formed and there was a release of polylysine and hygroscopic expansion [185].

In dental restorative fillings, bioglass released Ca and PO_4_ ions that filled the minute space between the tooth and restoration and had a toxic effect on bacteria present in the oral cavity, thus preventing bacterial accumulation and secondary caries and ultimately increased the restoration longevity [186]. It also enhanced the tooth enamel remineralization process due to its bioactivity [187]. In other studies, bioactive glass-based adhesives were applied to demineralized dentin, and dentin was observed to remineralize successfully [188,189,190]. Sauro et al. [191] probed the bonded-dentin interface healing response to applying pure Bioglass 45S5 and Zinc-polycarboxylated bioactive glass-based dental composite. The increased nano-mechanical properties, along with reduced micro-permeability at the dentin-bonded interface by therapeutic remineralization of imperfect mineral-depleted areas, were observed in the experimental dental composite. In another study [192], commercial bioactive glass (30% wt.) was incorporated in dental adhesive formulated with a mixture of bis-GMA + HEMA + acidic monomer (PMDM) and compared the properties with adhesive without bioactive glass. The micro-tensile bond strength was evaluated after 1- and 180-days storage in phosphate buffer solution (PBS). It was revealed that high values were attained in both groups after 1 day, with a cohesive mode of failures. Whereas, after 180 days of storage in PBS, the strength values decreased with mostly adhesive failure (66%). Further, the surface of the specimen was observed to bear ‘channeled’ dentinal tubules with unexposed collagen fibrils. Whereas adhesives with bioactive glass maintained strength values; both cohesive (43%) and mixed (40%) modes were observed. It was revealed that the dentin surface primarily protected by mineral crystals and residual resin embedded within a resin/collagen network. Sauro et al. [193] air abraded dentin surface with Bioglass and Bioglass/Polyacrylic solution (85:15 and 60:40 wt.%). After abrasion, the adhesive composite was applied to the dentin surface, and the micro-tensile bond strength was evaluated. It was observed that 100% Bioglass or PAA-15 wt.%/Bioglass did not affect the bonding capability of self-etching adhesives [193]. This technique might enhance the probability of bioactive glass particles infiltrating dentin tubules, which could subsequently lead to dentin surface remineralization, and could increase the bonding durability of the restorative materials [194]. It had been reported that bioglass-based dental composites showed superior behavior in terms of mechanical properties as compared to commercially available dental composites [195].

Biodentin™ (based on silicates and zirconium oxides) has been a widely accepted commercially available dentin replacement material since 2009 by Septodont [196]. The liquid components are calcium chloride and hydro-soluble polymer [197]. It is suggested that if bioactive liners apply under resin composite than glass ionomer liners, they would be well-tolerated biologically by the pulp tissue and advantageous clinically [198,199]. ACTIVA BIOACTIVE (Pulpdent Corp, Watertown, MA, USA) was marketed in 2013 as a novel resin-modified dental restorative material. Furthermore, it has proven a continuous passive diffusion of certain ions (P, F, and Ca) through the restorative material [200]. According to the manufacturers, this is the first commercially available bioactive dental composite with ionic resins [201]. ACTIVA is a flowable dual-cured self-adhesive composite [202]. The material possesses resin-modified glass ionomer (RM-GIC) properties with better resilience and other physical properties. A study showed that ACTIVA Bioactive exhibited comparable flexural fatigue and strength with flowable composites, and was significantly higher than conventional RM-GIC and glass ionomer cement (GIC) [203]. This material also showed the lowest bacterial microleakage compared to zinc phosphate cement [204]. It exhibited a significant rise in fluoride release after recharge at 1, 7, and 21 days when compared to conventional RM-GIC and GIC [205]. Further, it revealed increased surface wear compared to resin composites, RM-GIC, and GIC [206]. A laboratory study showed that elutes of this bioactive material were similar and more cytotoxic than calcium hydroxide and mineral trioxide aggregates, whereas ACTIVA elutes promoted biomineralization [207,208]. A study [209] compared the one-year clinical performance of ACTIVA Bioactive and SDR Bulkfill composites in primary molars and the results showed that the functional and esthetic properties of both materials were comparable. The biological properties of both composites were changed over time; however, the difference was not significant.

### 3.2. Endodontic Application

Endodontic material modifications had been applied to improve the success of root canal treatment (RCT). Using bioactive inorganic materials in endodontics could produce an apatite layer and improve interfacial adhesion of the material with root canal dentine, thus introducing a new concept in root canal filling [210]. The applications of root canal sealers based on bioactive inorganic materials has been found to generate more ideal sealer properties in various studies [211]. One desired property was long-lasting antibacterial activity, which could help to avoid re-contamination of the root canal system [212]. Bioactive glass had been utilized as a sealant or as a component of other sealers, such as resin-based sealers [213,214]. The commercially available bioactive glass-based root canal sealers are GuttaFlow Bioseal (GFB) (Coltè ne/Whaledent AG, Altstätten, Switzerland) and Nishika Canal Sealer Bioactive Glass (CS-BG) (Nippon Shika Yakuhin, Yamaguchi, Japan).

**Figure 12:**
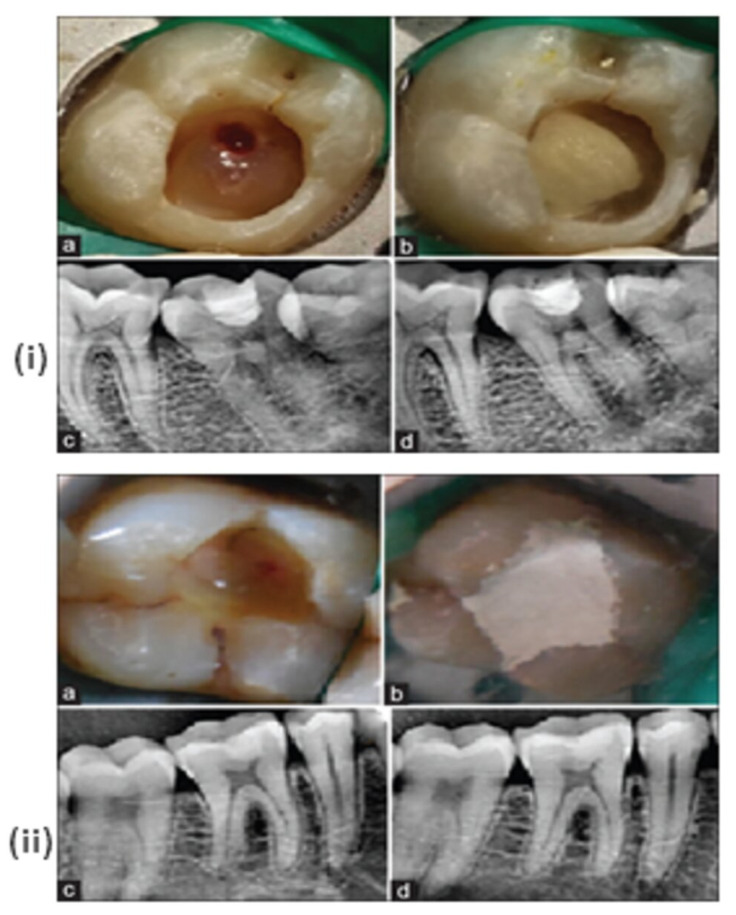
(**i**) (a) Exposure of pulp after caries excavation; (b) Placement of MTA over the exposed pulp; (c) Radiographic image showing placement of the MTA after pulp capping; (d) Follow-up radiograph after 6 months; (**ii**) (a) Exposure of pulp after caries excavation; (b) Placement of Biodentine over the exposed pulp; (c) Radiographic image showing placement of the Biodentine after pulp capping; (d) Follow-up radiograph after 6 months [215].

The use of tricalcium silicate in vital pulp therapy and root canal treatment has shown favorable outcomes in both adult and pediatric dentistry. Sankin apatite root sealer (ARS) (Dentsply Sankin, Tokyo, Japan) is a calcium phosphate-based sealer. ARS has three types, each type with a different powder composition, where type I has hydroxy-Sankin apatite and tricalcium phosphate, while iodoform is added in type II and III by 30% and 5%, respectively. A clinical study [215] showed that mineral trioxide aggregates and Biodentine were placed onto the pulp exposure sites and the results showed definite dentin-bridge formation at a 6-month follow-up (Figure 12). It is expected that both Biodentine and MTA tend to release calcium ions and have the ability to create alkaline pH, and subsequently the nucleation of ions and crystallization of the apatite layer on dentin surface. The formation of layer after 6 months could be due to the maturation of a carbonated-apatite phase [216].

The inorganic bioactive materials had been used extensively to repair perforation and pulpotomy. A study [217] reported the comparison of calcium phosphate cement and glass ionomer cement in the perforation depth and found no significant difference. Another study [218] reported that MTA had better results than IRM and amalgam for the repair of experimentally created root perforation. Guneser et al. [219] compared Biodentine and MTA and found better results for Biodentine as a perforation repair material. The repair of the perforated area depended on the release of calcium ions and formation of an apatite layer. Other materials, such as Endosequence and Bioaggregate, had been used for root repair and showed a precipitation of apatite crystals [220]. The calcium silicate-based materials had shown better results than calcium hydroxide in the treatment of exposed pulp. However, it is important to consider the composition of the calcium silicate-based materials since it can influence the properties such as setting time, radiopacity, and the causing pigments on the tooth [221]. Hydroxyapatite had also shown promising results when used as a direct pulp-capping agent. This material had a tendency to complete dentinal bridge formation. Biodentine and MTA are both reliable materials for treating root resorption due to their biocompatibility and ability to regenerate peri-radicular tissues [222].

### 3.3. Periodontal Applications

Periodontitis is considered one of the diseases of the periodontal tissues that involves loss of bone support and connective tissue, ultimately leading to tooth loss [223]. Different treatment planning has been considered for the treatment of periodontitis, including resorbable membranes (natural, synthetic), non-resorbable membranes [224], guided tissue regeneration (GTR) [225,226], guided bone regeneration (GBR), autologous membranes (developed through platelet concentrations) [227], and synthetic bone graft-perioglass, for treating periodontal infrabony defects [228,229].

**Figure 13:**
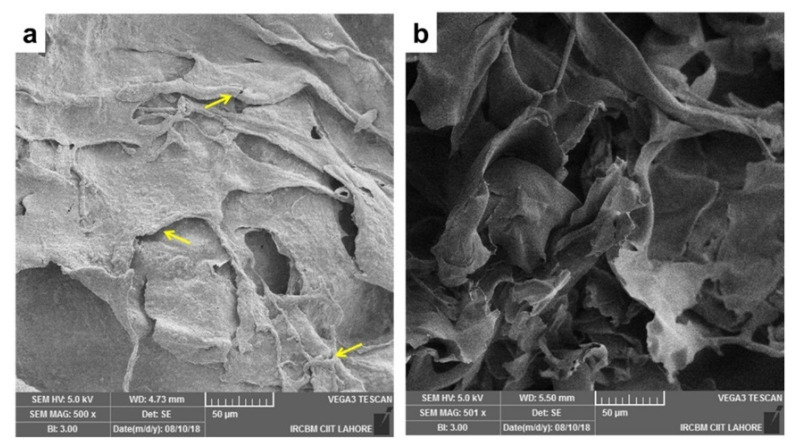
The SEM images showing the (**a**) lower surface and (**b**) upper surface of tri-layered membrane surface. The images are showing the presence of MC3T3-E1 pre-osteoblasts cells (yellow arrow) on surface [234].

**Figure 14:**
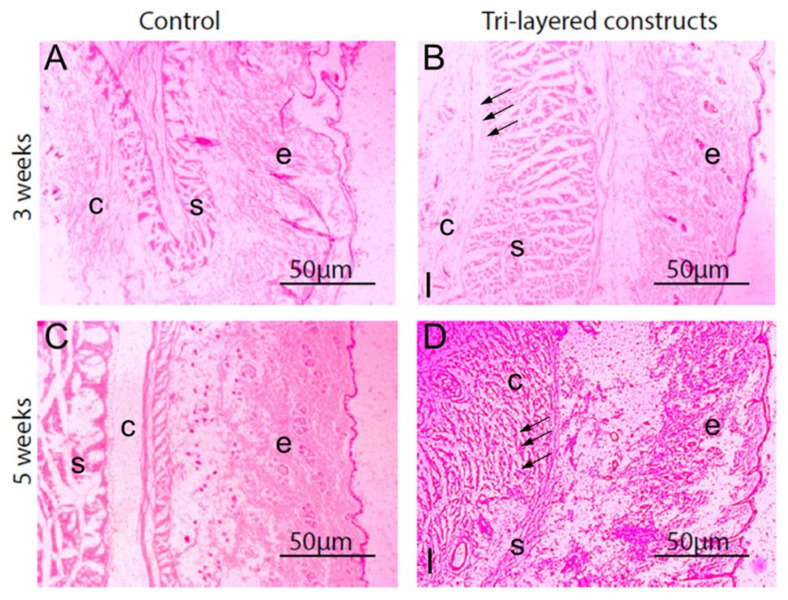
Histological images showing host inflammatory responses in subcutaneous tissue adjacent to functionally graded tri-layered membranes; (**A**) Tissue after three weeks of implantation with functionally graded membranes; (**B**) Peri-implant tissue (black arrows) indicates connective tissue without accumulation of inflammatory cells. Tissue without implants; (**C**) Peri-implant tissue after five weeks of implantation with functionally graded membranes; (**D**) Site of implantation indicates fibrous tissue (black arrows). Abbreviations: I, tissue near functionally graded chitosan membranes; c, connective tissue; e, epidermis; s, skeletal muscles [234].

The first paper on calcium phosphate biomaterial application in treating periodontal disease was published in 1975, which was followed by alveolar ridge augmentation, and HA compacted rod-like implant in the place of damaged roots [230,231,232]. β-TCP has wide applications in periodontics due to its chemical similarity with the cancellous bone and quick resorption. Data revealed its better periodontal attachment level; however, it is encapsulated by fibers leading to stunted new bone growth. The structural and chemical composition of β-TCP affects its characteristics [233]. The bioactive glass shows osteoinductive (induces osteogenesis) as well as osteoconductive behavior, i.e., permits bone growth on the surface [29,234]. Bioactive glasses are well known for increasing the periodontal ligament (PDL) level and curing tooth extraction sites by forming a carbonated HA in the tissue-graft interface [235]. They have the potential to promote cell attachment, proliferation, and differentiation [236]. Therefore, they act as carriers in tissue engineering [237], bone regeneration, and for production of synthetic bone substitutes. Various studies showed the incorporation of bioactive glass in experimental periodontal membranes (bi- and tri-layered membranes) and showed positive results in terms of cell compatibility (Figure 13) and regeneration (Figure 14) [238,239,240,241]. Perio-Glass is one item introduced to repair imperfections of the bony parts of the jaw and destructions caused by periodontal infections to the bone during the correction of malposition teeth [242].

### 3.4. Surgical Applications

**Figure 15:**
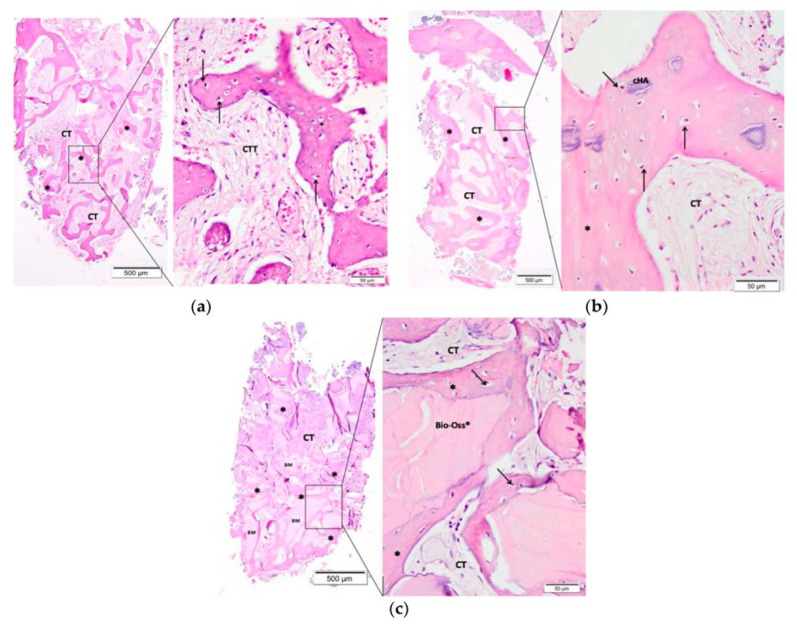
Microscopic images of the alveolar socket after 90 days of biomaterial implantation: (**a**) Control group (Clot), (**b**) carbonated hydroxyapatite group, and (**c**) Bio-Oss^®^ group. The small squares are displayed at 40-fold magnification adjacent to the figures with lower magnification. Connective tissue (CT/CIT); (*) new bone formation; osteoblast pavement (black arrow); carbonated hydroxyapatite (CHA) and Bio-Oss^®^ [248].

Scientists specializing in biomaterials’ health applications are exploring oral hard and soft tissue engineering with bioactive materials through activation of the body’s immune cells and different proteins. The approach is useful and has effectively improved the repair of tissues and managed the different conditions through surgery in dentistry [243]. Bioactive inorganic materials applied in dental surgery have undergone improvements over the years to what it is today. After tooth extraction, the empty socket is resorbed, leading to the shallowing of an alveolar ridge, which further causes denture retention problems and a decrease in facial height. The empty socket is filled with bioceramics that fill the gap and act as a part of the normal bone to prevent this issue. Osteoconductive and osteointegrative bioceramics have been commonly used for this application. They form close contact with the tissue and the graft [244]. The inorganic bioactive materials, such as calcium silicate, hydroxyapatite, bioactive glass, amorphous calcium phosphate, etc., have successfully restored maxillo-facial bone defect after root-end resection and removal of granulation tissue [245,246,247]. In a clinical trial study [248], carbonated hydroxyapatite and Bio-Oss^®^—Geistlich Biomaterials, Wolhuser, Switzerland) were placed after extraction, and a histological analysis of bone samples was completed after 90 days. Both groups showed the same bone density in both groups. The histological analysis (Figure 15) showed a larger amount of new bone along with the hydroxyapatite residual was observed with carbonated hydroxyapatite group. The Bio-Oss^®^ group also showed new bone formation; however, more residuals than carbonated hydroxyapatite. The less residuals of carbonated hydroxyapatite showed the tendency of quick resorption and release of more calcium and phosphate ions, subsequently helped in the formation of new bone. The factors associated with quick resorption of hydroxyapatite have been mentioned in earlier sections.

The use of bioactive glass in dental surgery is founded on its intrinsic features, making it suitable for different surgical applications in dentistry [249]. Surgical applications of bioactive glass include enamel bone regeneration, implant dentistry, and maxillofacial surgery. In maxillofacial surgery, bioactive glass has been found to help induce bone growth, both in quality and quantity, at an accelerated rate [250]. This makes the glass suitable for surgeries aimed at repairing defects in the teeth, as well as the jawbone and the repair of orbital floor fractures [251]. Compared to materials used in managing different dental problems, the bioactive glass mimics the natural dental tissues and enhances the effectiveness of the treatment of various health conditions involving dental tissues [46]. Some of the surgical applications of HA include reconstruction of periodontal bone defects and filling the bone defects following the loss of implants [252]. Additionally, the HA’s use was also evident in the benefits in the enhancement of the thickness of the atrophic alveolar ridges [243].

TCP had been applied in dental surgery based on its bioactive properties. Being a resorbable phase of calcium phosphate, the material was considered useful in supporting the growth of bones after surgery. TCP was particularly used as a material in bone grafting and as a bone material following the extraction of the teeth. The rationale for its usage was based on its efficacy in the maintenance of bone height and width. The use of TCP as osseous was also helpful in creating a healing environment after a surgical procedure in dentistry [253]. The preference for TCP in implant replacement in dental surgery was based on the predictability of the outcomes. The material showed improved integration into the natural bone, making it a preferred material. When mixed with a blood clot and added to the affected areas, TCP was postulated to enhance osteoblastic and fibroblastic activities, which were essential processes in replacing implants in dental surgery [253].

### 3.5. Implant Coatings

**Figure 16:**
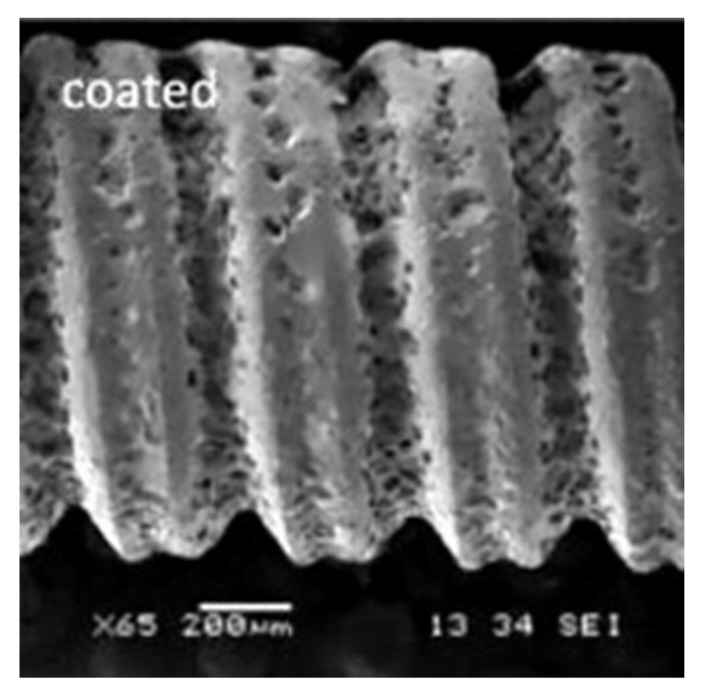
BG coating on a Ti_6_Al_4_V implant screw [254].

Implant surfaces after coating with bioactive inorganic materials showed favorable biocompatibility and enhanced bone healing performance [254]. The coatings had been completed on titanium and zirconia implants [255,256]. HA was used in dental implants based on its bioactive properties that mimic natural bone. Currently, HA was widely used in dental surgery as bone implants based on its chemical properties [257]. The material contained phosphate and calcium ions, which led to minimal toxicity when inserted as an implant following surgery. Additionally, when used in surgery, the resultant bone structure bonded directly to HA, a process that was mediated by a calcium-deficient layer that characterized the interface between the bone and the implant. Besides HA, bioactive glass had been used for implant coating, as shown in Figure 16. 

The bioactive glass and silicate coatings on implant surfaces showed enhanced bioactivity, the metabolic activity of osteoblasts, bone regeneration, and exhibited antibacterial properties [258,259,260,261]. Hu et al. substituted magnesium, zinc, and strontium ions for calcium in plasma-sprayed calcium silicate (Ca_2_MgSi_2_O_7_, Ca_2_ZnSi_2_O_7_, and Sr–CaSiO_3_) coatings [262]. It was found that the doping with these ions reduced the degradative behavior and improved the biological properties. The coating of silicates on metallic implant surfaces increased the surface roughness, bond strength, bactericidal activity, and altered the degradation rate [251].

Santos-Coquillat et al. [263] investigated the development of Plasma Electrolytic Oxidation coatings on cp titanium-containing calcium, phosphorous, and various combinations of magnesium, silicon, zinc, and fluorine. Their study showed that the coatings (15–27 μm) were generated in 300 s in mildly acidic, neutral, or alkaline transparent electrolytes stabilized by Na_2_EDTA. The bioactive elements incorporated into the coating from the electrolytes formed crystalline phases such as hydroxyapatite, silica, magnesium oxide, calcium fluoride, and calcium titanates. Many factors could influence the outcomes of bioactive material-coated implant surfaces, including biocompatibility, corrosion resistance, surface charge, topography, surface energy, wettability, etc. The enhanced wettability was related to improved biocompatibility [264,265]. The surface charge also determined the interaction of implant surface with plasma proteins, cells, and biofilm formation. Therefore, careful consideration must be applied to surface modification techniques that intended to manipulate the surface charge [266].

This review article provided comprehensive information related to bioactive inorganic materials and their applications in dentistry. The authors did not perform meta-analysis and did not focus on the systematic search as per the PRISMA guidelines. The authors did not provide detailed information about the change of phase-analysis of bioactive inorganic materials. The literature search related to the broad range of the bioactive materials, such as organic bioactive materials, was not included in this article. Furthermore, literature information about the detailed applications of ion-substituted hydroxyapatite and ion-doped bioactive glasses in relation to dentistry was not included. Moreover, a special focus on clinical trials and commercial materials based on bioactive inorganic materials should be given in future review articles.

## 4. Future Directions

This review shows the potential use of bioactive materials in dentistry; however, more intensive research is required to improve physical and mechanical properties without compromising the biological properties. Further, to make its use in load-bearing areas, the brittleness of these materials should be improvised. New challenging phases of improving the creative use of biomaterials in defective, aged, or diseased bone and oral hard tissues are increasing mechanical properties. However, there have been major improvements in bioceramics performed by key workers all over the world. The reported work (in vitro and in vivo) of bioceramics-based materials is mainly based on their application for hard tissue repair or regeneration; however, their application in the regeneration of soft tissues has been paid little attention. Recently, some in vitro studies have proven the ability of these materials to promote neocartilage formation and improve angiogenesis and recommend these for soft tissue wounds healing. The advantages of the novel biomaterials that have been developed so far are remarkable, whereby additional research is necessary to gain optimum benefits of the biomaterials clinically.

## 5. Conclusions

It is established that the bioactive inorganic materials are biocompatible and have strong regenerative potential. It is important to consider the physical and structural nature of the bioactive inorganic materials. The calcium phosphate-based materials have been used extensively in biomedical and dental applications. However, the Ca/P ratio, composition, and crystal structure can determine the properties of these materials. The materials, such as amorphous calcium phosphate, hydroxyapatite, tri-calcium phosphate, bioactive glass, and calcium silicates have been used to remineralize hard tissues, i.e., enamel and dentin, protection of dentin-pulp complex, reinforcing agents in resin-based composites, adhesives, and root canal sealers. Due to their ability to induce bone growth, hydroxyapatite and bioactive glass materials have been used for implant coating, periodontal regeneration, and in cranio-maxillo-facial surgery, whereby these materials showed promising results. It is well documented that the above-mentioned bioactive materials have excellent biological, exceptional biocompatible, and bioactive properties due to their similarity with human hard tissues. However, concern has been noted related to its mechanical properties. Therefore, more research is required to improve their physical and mechanical properties without decreasing their biological potential.

## Data Availability

Not applicable.
